# Investigating the Psychological Impact of COVID-19 among Healthcare Workers: A Meta-Analysis

**DOI:** 10.3390/ijerph17239096

**Published:** 2020-12-05

**Authors:** Kavita Batra, Tejinder Pal Singh, Manoj Sharma, Ravi Batra, Nena Schvaneveldt

**Affiliations:** 1 Department of Environmental and Occupational Health, University of Nevada, Las Vegas, NV 89119, USA; manoj.sharma@unlv.edu; 2 Office of Research, School of Medicine, University of Nevada, Las Vegas, NV 89102, USA; 3 Department of Family and Preventive Medicine, Division of Public Health, School of Medicine, University of Utah, Salt Lake City, UT 84108, USA; tp.singh@utah.edu; 4 Department of Information Technology and Testing Center of Excellence, Coforge, Atlanta, GA 30338, USA; ravi.batra@coforgetech.com; 5 Spencer S. Eccles Health Sciences Library, University of Utah, Salt Lake City, UT 84112, USA; nena.schvaneveldt@utah.edu

**Keywords:** COVID-19, SARS-COV-2, psychological, anxiety, depression, stress, post-traumatic stress syndrome, insomnia, burnout, fatigue

## Abstract

Previous meta-analyses were conducted during the initial phases of the COVID-19 pandemic, which utilized a smaller pool of data. The current meta-analysis aims to provide additional (and updated) evidence related to the psychological impact among healthcare workers. The search strategy was developed by a medical librarian and bibliographical databases, including Medline, Embase, CINAHL, PsycINFO, and Scopus were searched for studies examining the impact of the COVID-19 pandemic on the psychological health of healthcare workers. Articles were screened by three reviewers. Heterogeneity among studies was assessed by I^2^ statistic. The random-effects model was utilized to obtain the pooled prevalence. A subgroup analysis by region, gender, quality of study, assessment methods, healthcare profession, and exposure was performed. Publication bias was assessed by Funnel plot and Egger linear regression test. Sixty-five studies met the inclusion criteria and the total sample constituted 79,437 participants. The pooled prevalence of anxiety, depression, stress, post-traumatic stress syndrome, insomnia, psychological distress, and burnout was 34.4%, 31.8%, 40.3%, 11.4%, 27.8%, 46.1%, and 37.4% respectively. The subgroup analysis indicated higher anxiety and depression prevalence among females, nurses, and frontline responders than males, doctors, and second-line healthcare workers. This study highlights the need for designing a targeted intervention to improve resilience and foster post-traumatic growth among frontline responders.

## 1. Introduction

In December 2019, a novel coronavirus originated in Wuhan (China), which was later identified by the International Committee on Taxonomy of Viruses (ICTV) as SARS-CoV-2 causing the disease COVID-19 [1]. The spread of the virus was rampant, with the cases spiraling up to nearly 148,838 in March, which led to COVID-19 being designated as a pandemic by the World Health Organization [WHO] on 11 March 2020 [2]. Undoubtedly, pandemics have a long-standing history of impacting physical and mental health across all population groups, of which healthcare workers (HCWs) bear a disproportionate burden [3]. During Severe Acute Respiratory Syndrome (SARS) and Middle East Respiratory Syndrome (MERS) outbreaks, a sizable proportion of the HCWs experienced anxiety, emotional distress, and post-traumatic stress disorder (PTSD, aka fatigue battle syndrome) [4,5,6]. These adverse psychological outcomes persisted until 1–3 years in the post-pandemic periods [6]. Following the aggressive course of transmission, the COVID-19 pandemic has taken a firm grip worldwide and has surpassed the historical outbreaks in generating extraordinary amounts of pressure and psychiatric morbidities among healthcare workers (HCWs) [3,6,7,8,9]. Physical and emotional exhaustion associated with managing large volumes of COVID-19 cases, shortage of personal protective equipment (PPE), risk of nosocomial infections, and fear of secondary transmission to family members, feelings of being rejected by others, and social isolation make frontline HCWs more vulnerable to the psychological corollaries of the COVID-19 pandemic [7,8,10]. Similar to previous outbreaks, the stress induced by this bio-disaster (COVID-19) has the potential to develop into PTSD [11,12,13]. In one Japan-based study, stress associated with deployment activities among the healthcare workforce was investigated [11]. The symptoms of PTSD were reported among Disaster Medical Assistance Team (DMAT) members, who were deployed to analyze, manage, and contain the transmission of COVID-19 on a Diamond Princess Cruise ship during the initial phases of the pandemic [11,12]. PTSD has already been cited as the main psychiatry disorder associated with disaster-related experiences or activities, especially among those being on the front lines of the battle against the pandemic [13]. In the wake of the COVID-19 pandemic, frontline responders are continuously working to meet the heavy healthcare demands and are exposed to higher levels of psychiatric morbidities. It is likely that these psychiatric issues will take a chronic course and will translate into PTSD in the repairing phases of the pandemic. Therefore, it is critical to assess the psychological impact among HCWs throughout the evolution of the COVID-19 pandemic to design early interventions to improve psychological outcomes. Several qualitative and quantitative studies have been conducted to explore all dimensions of the psychological spectrum among HCWs and provided valuable insights [11,13,14,15].

Previous meta-analyses investigated the psychological impact on healthcare populations during the early episodes of this bio-disaster and utilized a smaller pool of data [14,15]. One (single- arm) meta-analysis of observational studies included 13 studies [16,17,18,19,20,21,22,23,24,25,26,27,28,29,30,31,32,33,34,35,36,37,38,39,40,41] with a sample of 33,062 participants [14]. This study was based on a literature search on April 17, 2020. Another meta-analysis attempted to expand the evidence (by adding 12 more studies) [29,30,31,32,33,34,35,36,37,38,39,40] by comparing the psychological impact among the general population, healthcare workers, and patients with preexisting conditions [15]. This study included a total of 19 studies [16,17,18,19,20,21,29,30,31,32,33,34,35,36,37,38,39,40,41] (based on a search on May 25, 2020), and the overall prevalence of psychological indicators among healthcare workers with no further distinction based on exposure (i.e., frontline and second-line workers) were reported [15]. With the rapid evolution of pandemic and increased number of hospitalizations, frontline HCWs are experiencing unprecedented emotional and psychological challenges [15,33,34,35,36,37]. Along the course of the pandemic, new studies (*n* = 38) have rapidly been conducted in different parts of the world, [6,42,43,44,45,46,47,48,49,50,51,52,53,54,55,56,57,58,59,60,61,62,63,64,65,66,67,68,69,70,71,72,73,74,75,76,77,78,79,80,81], which will provide additional insight into the existing literature. Additionally, it is imperative to make continuous efforts in collecting and synthesizing more data until the full picture of the psychological toll on healthcare workers emerges. Therefore, the purpose of this meta-analysis is to provide updated evidence (based on search prior to July 27, 2020, with 40 additional studies) across 24 countries to investigate the psychological impact of the COVID-19 pandemic on HCWs, with further stratification to the level of COVID-19 exposure.

## 2. Materials and Methods

### 2.1. Protocol Registration

To conduct a meta-analysis on studies investigating the psychological impact of the COVID-19 pandemic among health care workers, we followed the Preferred Reporting Items for Systematic Reviews and Meta-Analyses [PRISMA] guidelines [82]. This study protocol was registered in the International Prospective Register of Systematic Reviews (PROSPERO: CRD42020205824; https://www.crd.york.ac.uk/PROSPERO/).

### 2.2. Databases and Search Strategy

Information retrieval was conducted by a medical librarian (N.S.) on July 27, 2020. Our primary database was MEDLINE. The MEDLINE strategy was developed, then peer-reviewed by colleagues prior to being translated to other databases. The full search strategy is available in Appendix A. We searched MEDLINE (Ovid) 1946–2020, Embase (embase.com) 1974–2020, CINAHL Complete (EBSCOhost) 1937–2020, PsycINFO (EBSCOhost) 1872–2020, and Scopus (scopus.org) 1970–2020. For preprints, we searched medRxiv (https://www.medrxiv.org/) and SSRN’s COVID-19 Research Topic (https://www.ssrn.com/index.cfm/en/). The references of included publications and previous reviews were also assessed to identify additional studies.

### 2.3. Eligibility Criteria

Quantitative and observational studies based on original research examining the impact of the COVID-19 pandemic on the psychological health of HCWs were included. Studies which met the following criteria were included: (1) directly related to the context of the impact of the COVID-19 pandemic and reported any or a combination of the psychological outcomes, including anxiety, depression, stress, psychological distress, insomnia, and sleep disturbances; (2) non-interventional; (3) conducted on a healthcare population; (4) published in the English language; (5) published between December 1, 2019, to July 27, 2020; (6) used validated assessment methods or survey instruments to record the psychological outcomes; (7) available as full-texts. We excluded studies if they: (1) were irrelevant to the exposure (COVID-19) and the psychological outcomes; (2) were interventional; (3) were conducted on a general population; (4) reported insufficient data with unclear methods; (5) were not in the English language; (6) were conducted before December 1, 2019, and after July 27, 2020; (7) utilized qualitative or mixed methods; (8) did not use validated survey tools; (9) were case reports, reviews, editorials, duplicates, abstracts/poster-only records, animal studies, or biochemical studies.

### 2.4. Selection Process

**Figure 1:**
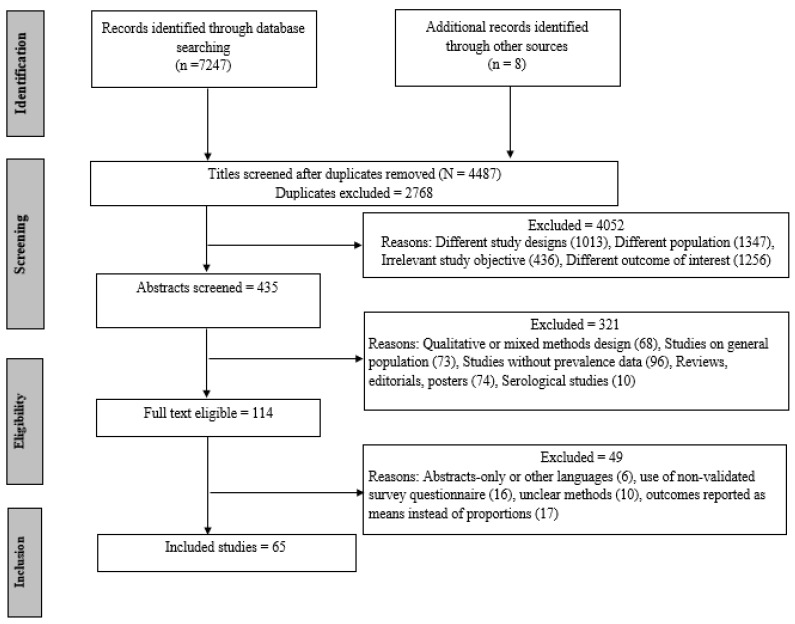
PRISMA (Preferred Reporting Items for Systematic Reviews and Meta-Analysis) flow diagram detailing the disposition of screened, included, and excluded records.

Results were first exported into an EndNote library for deduplication and then imported to Rayyan for screening. Three reviewers (K.B., T.P.S., and R.B.) performed screening independently and did not know each other’s decisions. All articles first underwent title screening, and then advanced to abstract screening, if deemed relevant. Then, the full texts of the selected abstracts were assessed to determine the eligibility of articles for inclusion. If an article was not included, the reason for exclusion was listed (Figure 1, PRISMA). If there were any disagreements, then the senior investigator (M.S.) evaluated the article, and a consensus was achieved through discussion.

### 2.5. Data Extraction

Full texts of eligible articles were obtained for data abstraction. Three independent reviewers (K.B., T.P.S., and R.B.) abstracted all studies for potential inclusion using a customized data abstraction form. Inconsistencies between the three reviewers were adjudicated by a fourth, independent reviewer (M.S.). The data elements included information about the author with year, study title, study location, gender proportion, categories of healthcare occupations (if available), sample size, assessment methods with the cut-off scores, and the prevalence of anxiety, depression, stress, psychological distress, insomnia, and impaired sleep quality. Data related to each study were verified for accuracy; any discrepancies were resolved through a discussion between the reviewers. We attempted to contact corresponding authors of included records to obtain additional information when there was uncertainty about study characteristics and data points.

### 2.6. Assessment of Bias Risk

The quality assessment was performed by utilizing the National Institutes of Health (NIH) quality assessment tool (https://www.nhlbi.nih.gov/health-topics/study-quality-assessment-tools) to assess the quality of the studies. Two reviewers (K.B. and T.P.S.) independently evaluated the risk of bias and quality of the studies and rated them according to the tool’s dictionary and guidelines. After assessing all the study components, the overall rating was determined using the criteria listed in the tool. Based on the number of “yes” answers, a rating of good (7–9), medium (4–6), or poor (≤3) was assigned to each study (Appendix B). Disagreements related to quality scores were resolved through a discussion among the reviewers, and a consensus was achieved upon the final judgement offered by the senior investigator (M.S.). The rating of two reviewers was compared, and the inter-rater agreement was calculated using Microsoft Excel.

### 2.7. Statistical Analysis

The overall prevalence and 95% confidence intervals of psychological outcomes were pooled using the Comprehensive Meta-Analysis Package (CMA version 3.0, Englewood, NJ, USA). The primary effect measure was the proportions of events, such as anxiety, depression, stress, psychological distress, insomnia, and impaired sleep quality. Due to methodologic variations and sample diversity across studies, the random-effects model was used to extract the pooled estimate [83]. Heterogeneity was assessed by the I^2^ statistic, which measures the percentage of variance resulting from true differences in the effect sizes rather than the sampling error. Substantial heterogeneity [84] was defined as I^2^ > 50%. Subgroup analysis (by potential sources of heterogeneity) was conducted according to the categorical moderating variables: country (China vs. other countries), gender, continent (Asia vs. other continents), quality of the study (good/medium), assessment methods, health care profession (doctors vs. nurses), type of exposure (high risk or frontline vs. low risk or second-line), and severity of psychological symptoms (mild/moderate/severe). Sensitivity analysis was conducted to identify studies which may severely affect the pooled prevalence. Funnel plot and Egger linear regression test was used to assess publication bias [85]. Significant level was set as two-sided and *p* < 0.05.

## 3. Results

### 3.1. Study Screening

Our systematic search yielded 7255 potentially relevant papers (Figure 1), out of which 2768 duplicate studies were removed. The titles of the remaining 4487 records were screened, and 4052 studies were excluded because the studies were conducted on non-healthcare population (*n* = 1347), had a different outcome of interest (*n* = 1256), different study designs, including case series, reviews, perspectives or opinions, and interventional studies (*n* = 1013), and were irrelevant to the study’s objective (*n* = 436). This resulted in 435 papers, which were advanced to abstract screening. A total of 321 papers were excluded after abstract screening because they were qualitative or mixed studies (*n* = 68), studies on a general population (*n* = 73), studies without prevalence data (*n* = 96), serological studies (10), or they were published as editorials, posters, or reviews (*n* = 74). Full-text screening of the remaining 114 papers generated 65 articles, which were included in the final review. Forty-nine articles were excluded in the final step because of following reasons: they were abstracts-only or in other languages (*n* = 6), used non-validated questionnaires (*n* = 16), had an unclear methodology or of low quality (*n* = 10), or had outcomes reported as means instead of the number of cases/or proportions (*n* = 17) (Figure 1).

### 3.2. Study Quality

Twenty-nine studies were of good quality [16,17,18,19,24,25,26,27,28,31,34,39,41,42,44,46,53,54,57,59,60,61,63,64,66,68,71,79,80] (score range 7–9) and thirty-six studies were of medium quality [6,20,21,23,27,29,30,32,33,35,36,37,38,43,45,47,48,49,50,51,52,55,56,58,62,65,67,69,70,72,73,74,76,77,78,81] (score range: 4–6). The quality scores of the included study evaluation as assessed using the National Institutes of Health quality assessment tool are summarized (Table A2 in Appendix B). The ratings of two reviewers (K.B. and T.P.S.) were averaged, and the inter-rater agreement was 84.5.

### 3.3. Study Characteristics

After deduplication and screening, 65 studies (Table A3 in Appendix C) [6,16,17,18,19,20,21,23,24,25,26,27,28,29,30,31,32,33,34,35,36,37,38,39,41,42,43,44,45,46,47,48,49,50,51,52,53,54,55,56,57,58,59,60,61,62,63,64,65,66,67,68,69,70,71,72,73,74,75,76,77,78,79,80,81] with a total of 79,437 participants were included in the analysis. Among them, 51 were from Asia (31 from China, 4 from India, 1 from Singapore, 1 from India and Singapore, 3 from Iran, two from Pakistan, 2 from Jordan, 1 from Bahrain, 1 from Hong Kong, 1 from Israel, 1 from Nepal, 1 from Oman, 1 from Saudi Arabia, and 1 from South Korea), 10 were from Europe (3 from Italy, 4 from Turkey, 1 from Switzerland, 1 from Serbia, and 1 from Ireland), 2 were from South America (1 from Argentina, Brazil, Chile and Mexico, and one from Brazil alone), and 2 were from North America. All the studies were cross-sectional and reported at least one psychological outcome (if not all) among HCWs in the context of the COVID-19 pandemic. The median number of individuals per study was 582 (range: 37 to 11,118) with a median male proportion of 25% (range: 0 to 96%) and a median response rate of 20.0% (range: 10.2% to 100%). Nearly 3/4th of the sample was female (*n* = 57,244; 72%). In terms of occupation distribution, nurses constituted nearly 45.7% (*n* = 36,315), followed by physicians or doctors, forming 1/4th (*n* = 19,287) of the entire sample. Remaining professions include allied health staff, laboratory specialists, anesthetist technicians and general technicians, physical therapists, pharmacists, dental professionals, etc.

### 3.4. Meta-Analysis

#### 3.4.1. Anxiety Prevalence

**Figure 2:**
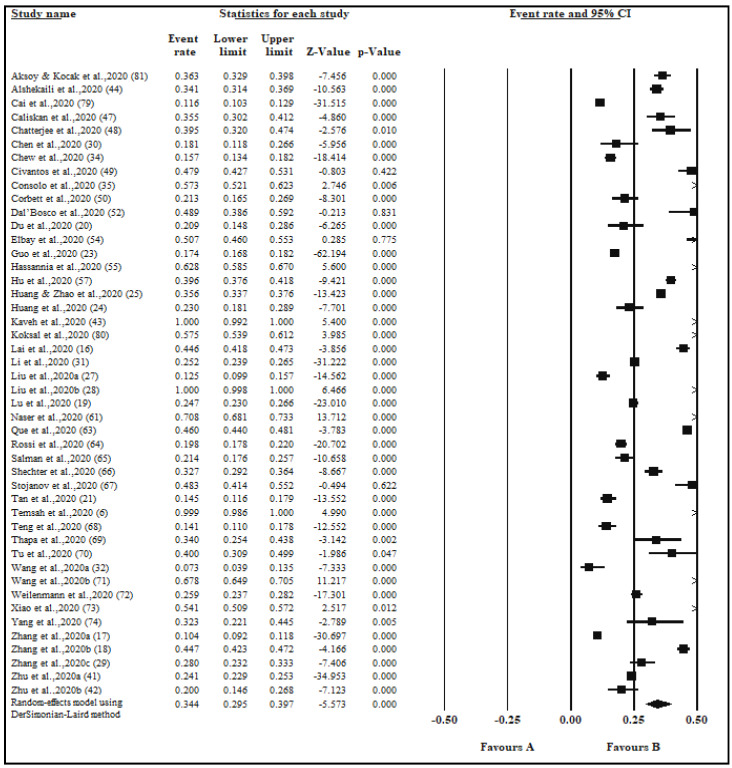
Forest plot for the studies on the prevalence of anxiety among healthcare workers. The squares and horizontal lines correspond to the study-specific event (anxiety) rates and 95% confidence intervals (CIs). The diamond represents the pooled prevalence and 95% CIs of the overall population. The overall pooled anxiety using a random effects DerSimonian-Laird method was 34.4% (95% CI: 29.5–39.7).

The pooled prevalence of anxiety in 46 studies with a sample size 51,596 was 34.4% (Table 1, Figure 2). The pooled prevalence of anxiety among good quality studies (*n* = 22) was 31.2% compared to 38.1% among medium quality studies (*n* = 24) (Table 1). The pooled prevalence of anxiety in the continent of Asia across 34 studies was 32.7% compared to 39.3% found in studies among other continents. Twenty-two studies were conducted in China and had a pooled prevalence of 28.5% as opposed to the 40.4% (Table 1) pooled prevalence across studies (*n* = 24) conducted in other countries. The Generalized Anxiety Disorder survey questionnaire was used across 19 studies and a pooled prevalence of 36.8% was found (Table 1). Gender data were available in seven studies with a pooled prevalence of 46.9% for females and 44.2% for males. In groups by healthcare professions, the pooled prevalence was higher in nurses compared to doctors (39.3% vs. 32.5%). Anxiety by exposure with a pooled prevalence of 39.8% among frontline HCWs compared to 27.1% prevalence among second-line HCWs. Levels of anxiety with the highest pooled prevalence of 60.3% related to mild symptoms, followed by a 26.0% prevalence of moderate symptoms and a prevalence of only 14.3% for severe symptoms (Table 1).

#### 3.4.2. Depression Prevalence

**Figure 3:**
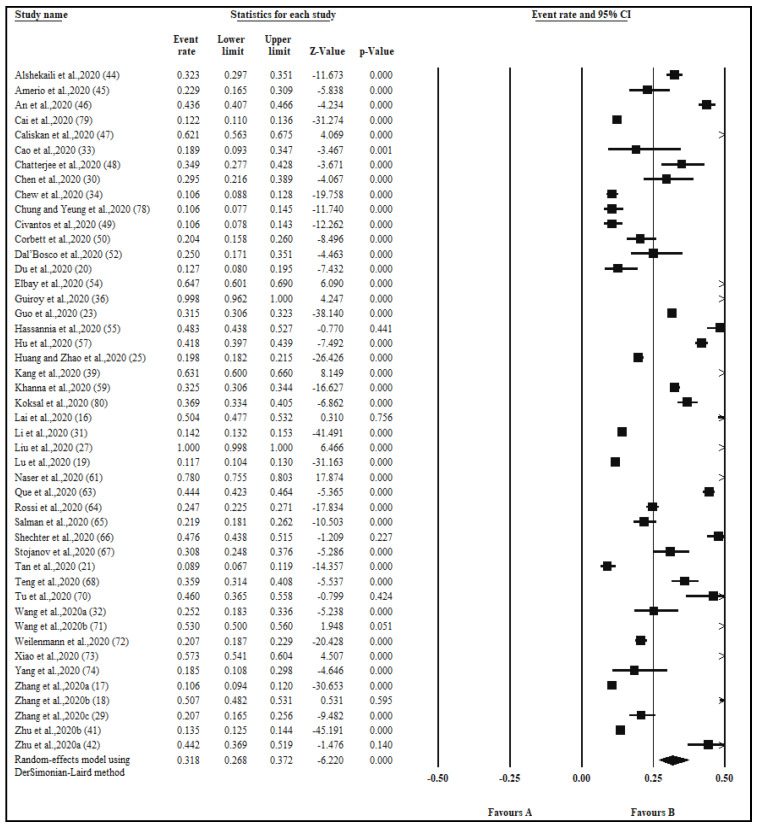
Forest plot for the studies on the prevalence of depression among healthcare workers. The squares and horizontal lines correspond to the study-specific event (anxiety) rates and 95% confidence intervals (CIs). The diamond represents the pooled prevalence and 95% CI of the overall population. The overall pooled depression using a random effects DerSimonian-Laird method was 31.8% (95% CI: 26.8–37.2).

The pooled prevalence of depression in 46 studies with a sample size 53,164 was 31.8% (Table 2, Figure 3). The pooled prevalence of depression among good quality studies was 35.1%, compared to 28.6% among medium quality studies (Table 2). The pooled prevalence of depression in the continent of Asia was 30.8% compared to 35.0% found in other continents. Twenty-three studies were conducted in China and had a pooled prevalence of 33.2% as opposed to the 30.4% pooled prevalence across studies conducted in other countries (*n* = 23). The Patient Health Questionnaire (PHQ) was used across 25 studies and a pooled prevalence of 29.7% was found. Gender data were available in seven studies, with a pooled prevalence of 43.4% for females and 40.9% for males. In the healthcare profession groups, examined in nine studies, the pooled prevalence of depression was higher in nurses compared to doctors (42.4% vs. 39.1%). Depression by exposure was reported in six studies with a pooled prevalence of 23.6% among frontline healthcare workers compared to 19.6% prevalence among second-line healthcare workers. Levels of depression were reported in 17 studies, with the highest pooled prevalence of 57.6% related to mild symptoms, followed by a 27.9% prevalence of moderate and only 10.4% of severe symptoms (Table 2).

#### 3.4.3. Stress Prevalence

**Figure 4:**
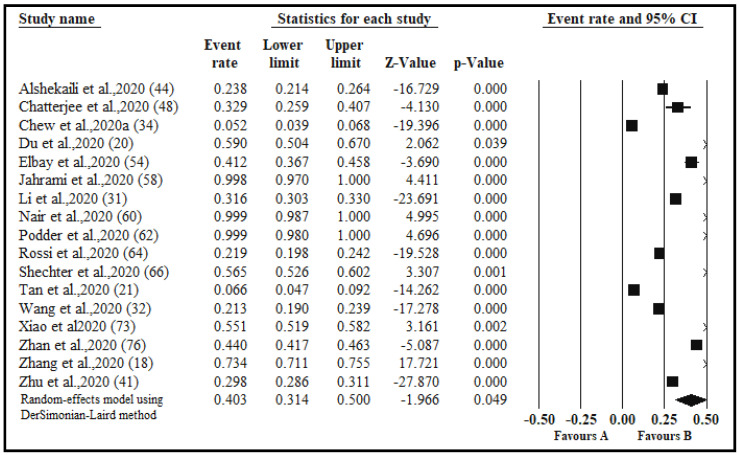
Forest plot for the studies on the prevalence of stress among healthcare workers. The squares and horizontal lines correspond to the study-specific event (anxiety) rates and 95% confidence intervals (CIs). The diamond represents the pooled prevalence and 95% CIs of the overall population. The overall pooled stress using a random effects DerSimonian-Laird method was 40.3% (95% CI 31.4–50.0).

The pooled prevalence of stress in 17 studies with a sample size of 16,235 was 40.3% (Table 3, Figure 4). The pooled prevalence of stress among good quality studies (*n* = 9) was 37.3% compared to 45.7% among medium quality studies (*n* = 8). The pooled prevalence of stress in the continent of Asia across 14 studies was 41.3% compared to 38.8% found in other continents. Seven studies were conducted in China and had a pooled prevalence of 44.2% as opposed to the 37.1% pooled prevalence in studies conducted in other countries (*n* = 10). The Perceived Stress Scale (PSS) was used across eight studies and a pooled prevalence of 61.4% was found. Levels of stress were reported in six studies, with the highest pooled prevalence related to moderate symptoms of 52.3%, followed by a 25.8% prevalence of mild and 18.9% of severe symptoms (Table 3). For stress, subgroup analyses by gender, healthcare occupations, and risk exposure were not conducted due to inadequate data.

#### 3.4.4. Prevalence of Insomnia and Impaired Sleep Quality

**Figure A1:**
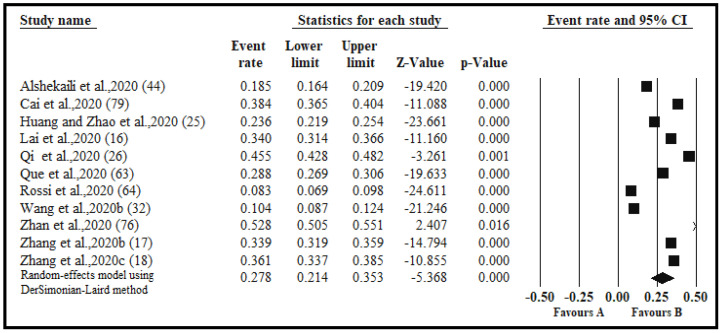
Forest plot for the studies on the prevalence of insomnia among healthcare workers. The squares and horizontal lines correspond to the study-specific event (anxiety) rates and 95% confidence intervals (CIs). The diamond represents the pooled prevalence and 95% CI of the overall population. The overall pooled insomnia using a random effects DerSimonian-Laird method was 27.8% (95% CI: 21.4–35.3).

**Figure A2:**
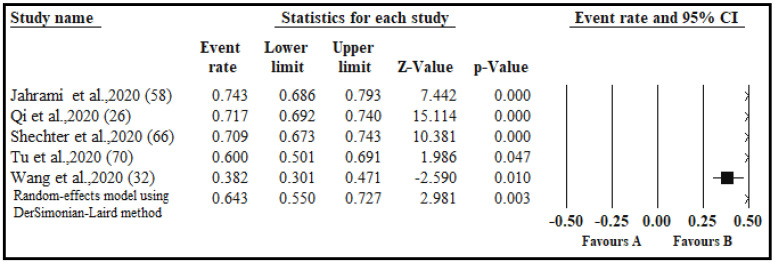
Forest plot for the studies on the prevalence of impaired sleep quality among healthcare workers. The squares and horizontal lines correspond to the study-specific event (anxiety) rates and 95% confidence intervals (CIs). The diamond represents the pooled prevalence and 95% CI of the overall population. The overall pooled impaired sleep quality using a random effects DerSimonian-Laird method was 64.3% (95% CI: 55.0–72.7).

Under the random effects model, the overall prevalence of insomnia in a sample size of 18,546 was 27.8% (95% CI: 21.4–35.3, I^2^ = 98.1%; *p* < 0.001; Figure A1 in Appendix C across 11 studies [16,17,18,25,26,32,44,63,64,76,79]. The insomnia severity index (ISI) was used across eight studies [17,18,26,32,44,63,64,79] and a pooled prevalence of 62.8 (95% CI: 44.8–77.9; I^2^ = 98.2%; *p* value < 0.001) was found. In groups by healthcare professions, three studies were included [16,18,63]. The pooled prevalence of insomnia was slightly higher in nurses compared to doctors (42.4% vs. 39.1%). The quality of sleep was assessed in five studies [26,32,58,66,70]. The overall prevalence of impaired sleep quality in a sample size of 2443 was 64.3% (95% CI:55.0–72.7, I^2^ = 93.1%; *p* < 0.001; Figure A2 in Appendix C). Subgroup analysis for insomnia by gender and exposure was not conducted due to inadequate data.

#### 3.4.5. Other Psychological Indicators

**Figure A3:**
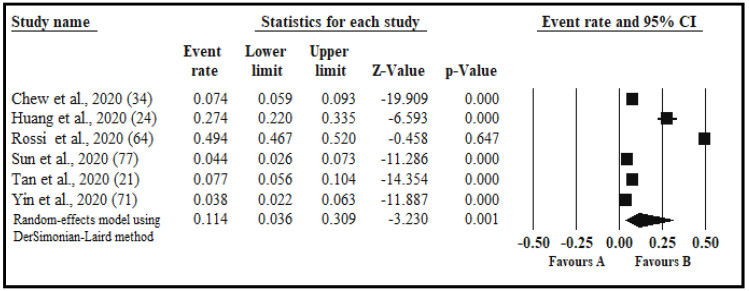
Forest plot for the studies on the prevalence of Post-traumatic stress syndrome (PTSD) among healthcare workers. The squares and horizontal lines correspond to the study-specific event (anxiety) rates and 95% confidence intervals (CIs). The diamond represents the pooled prevalence and 95% CI of the overall population. The overall pooled PTSD using a random effects DerSimonian-Laird method was 11.4% (95% CI: 3.6–30.9).

**Figure A4:**
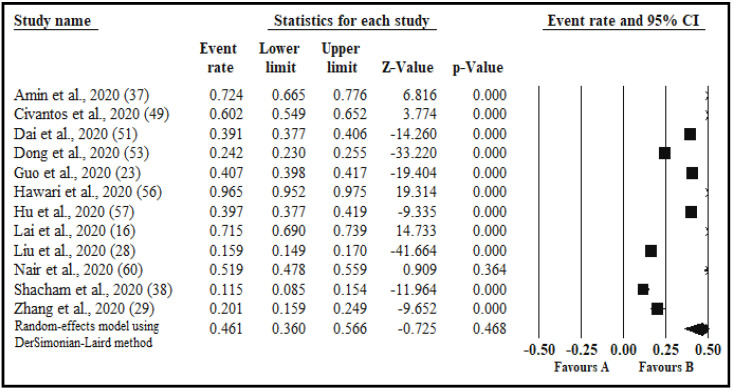
Forest plot for the studies on the prevalence of psychological distress among healthcare workers. The squares and horizontal lines correspond to the study-specific event (anxiety) rates and 95% confidence intervals (CIs). The diamond represents the pooled prevalence and 95% CI of the overall population. The overall pooled psychological distress using a random effects DerSimonian-Laird method was 46.1% (95% CI: 36.0–56.6).

**Figure A5:**
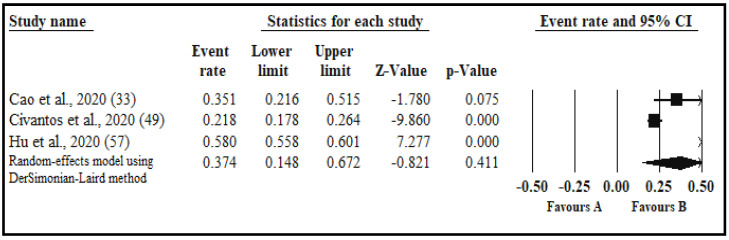
Forest plot for the studies on the prevalence of burnout among healthcare workers. The squares and horizontal lines correspond to the study-specific event (anxiety) rates and 95% confidence intervals (CIz). The diamond represents the pooled prevalence and 95% CI of the overall population. The overall pooled burnout using a random effects DerSimonian-Laird method was 37.4% (95% CI: 14.8–67.2).

Post-traumatic stress disorder (PTSD) was assessed in six studies [21,24,34,64,71,77]. Under the random effects model, the overall prevalence of PTSD in a sample size of 3676 was 11.4% (95% CI: 3.6–30.9; I^2^ = 99.2% *p* < 0.001; Figure A3 in Appendix C). Psychological distress was reported in 12 studies [16,23,28,29,37,38,49,51,53,56,57,60]. Under the random effects model, the overall prevalence of psychological distress in a sample size of 30,963 was 46.1% (95% CI: 36.0–56.6; I^2^ = 99.6%; *p* < 0.001; Figure A4 in Appendix C). Burnout was assessed in three studies [33,49,57] and the overall prevalence in a sample size of 2487 was 37.4% (95% CI: 14.8–67.2; I^2^ = 98.6%; *p* < 0.001; Figure A5 in Appendix C).

#### 3.4.6. Publication Bias

**Figure A6:**
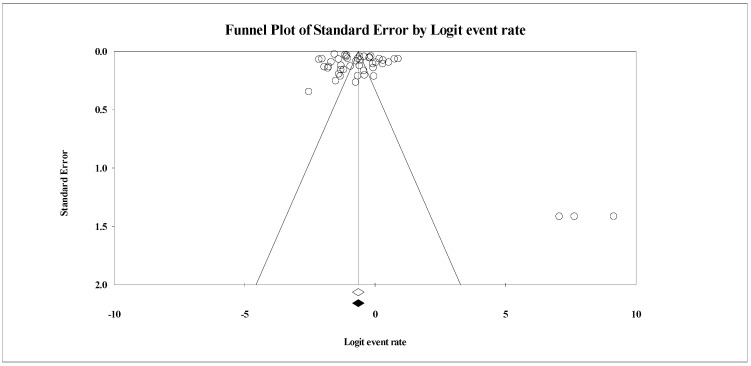
Funnel plot for studies on the prevalence of anxiety (Egger test: P = 0.15; Begg test: P = 0.90). The vertical solid line represents the summary effect estimates.

**Figure A7:**
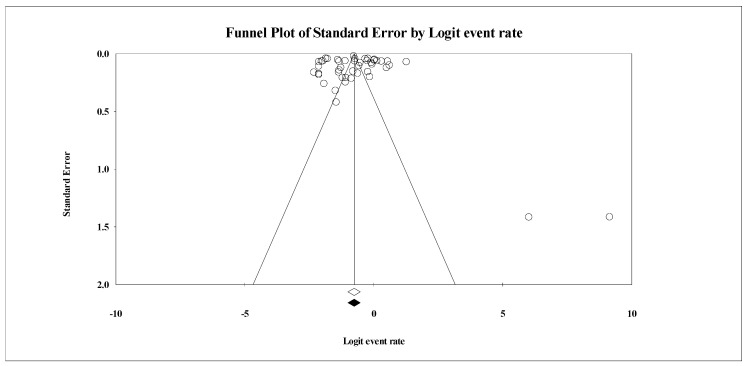
Funnel plot for studies on the prevalence of depression (Egger test: P = 0.90; Begg test: P = 0.64). The vertical solid line represents the summary effect estimates.

**Figure A8:**
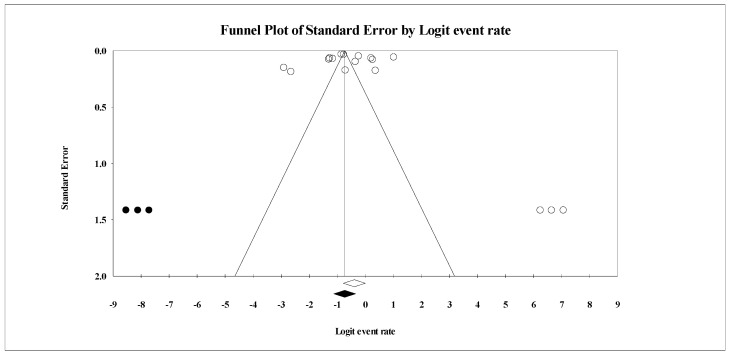
Funnel plot for studies on the prevalence of stress (Egger test: P = 0.69; Begg test: P = 0.86). The vertical solid line represents the summary effect estimates.

**Figure A9:**
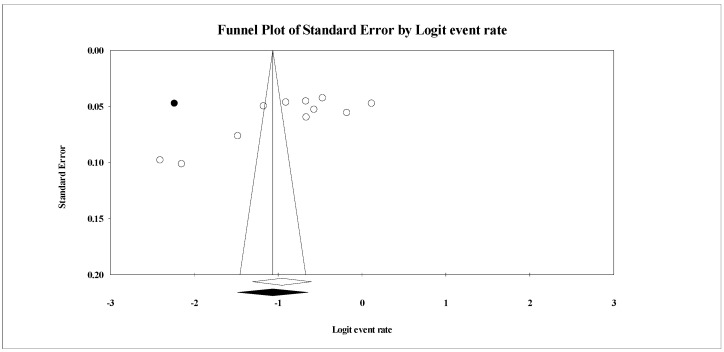
Funnel plot for studies on the prevalence of insomnia (Egger test: P = 0.01; Begg test: P = 0.03). The vertical solid line represents the summary effect estimates.

**Figure A10:**
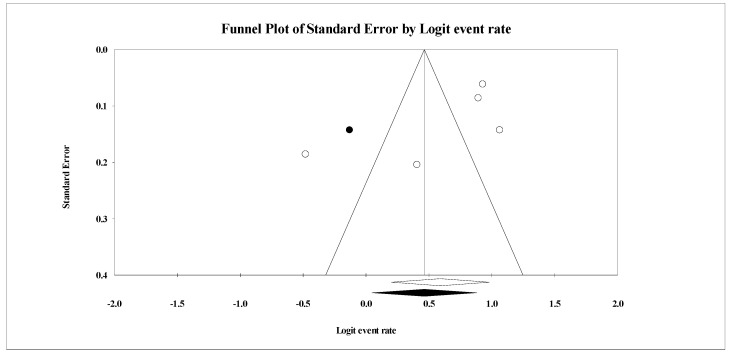
Funnel plot for studies on the prevalence of impaired sleep quality (Egger test: P = 0.22; Begg test: P = 0.22). The vertical solid line represents the summary effect estimates.

**Figure A11:**
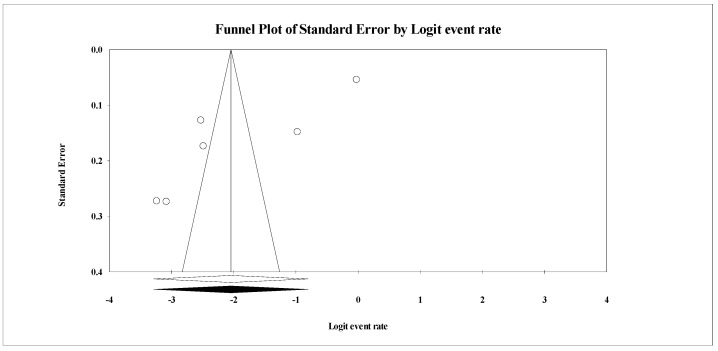
Funnel plot for studies on the prevalence of PTSD (Egger test: P = 0.22; Begg test: P = 0.90). The vertical solid line represents the summary effect estimates.

**Figure A12:**
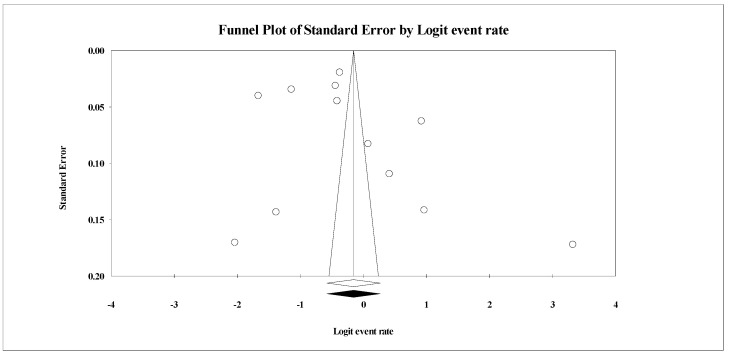
Funnel plot for studies on the prevalence of psychological distress (Egger test: P = 0.45; Begg test: P = 0.73). The vertical solid line represents the summary effect estimates.

**Figure A13:**
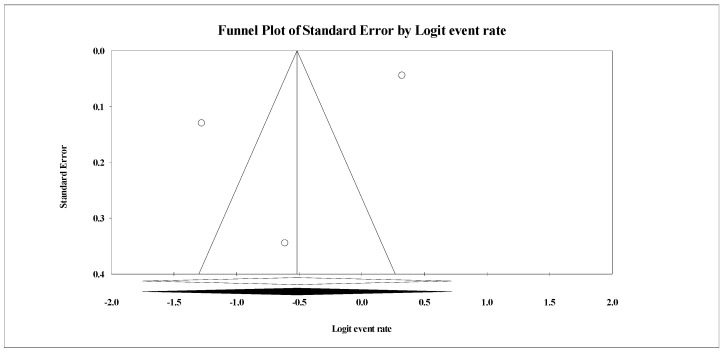
Funnel plot for studies on the prevalence of burnout (Egger test: P = 0.47; Begg test: P = 0.60). The vertical solid line represents the summary effect estimates.

The publication bias was assessed with Egger’s test indices. As indicated by the *p* values for the prevalence of anxiety (Egger test: *p* = 0.15), depression (*p* = 0.90), stress (*p* = 0.69), insomnia (*p* = 0.01), impaired sleep quality (*p* = 0.22), PTSD (*p* = 0.22), psychological distress (*p* = 0.45), and burnout (*p* = 0.47) (Figure A6, Figure A7, Figure A8, Figure A9, Figure A10, Figure A11, Figure A12, Figure A13 in Appendix C), the publication bias was insignificant for all the psychological outcomes, except insomnia.

## 4. Discussion

This pooled analysis included a large data sample of studies (*n* = 65) with 79,437 participants, compared to previous meta-analyses that included 13–19 studies [14,15]. Notably, to our knowledge, this study is the largest to evaluate the psychological impact of COVID-19 among HCWs. Moreover, we extended the existing evidence by including other psychological outcomes of stress, psychological distress, burnout, and impaired sleep quality. The findings suggest that the overall prevalence of anxiety, depression, stress, insomnia was 34.4%, 31.8%, 40.3%, and 27.8%, respectively. Compared to previous meta-analyses [14,15], we report a higher prevalence for anxiety (34.4% vs. 23.2%–26.0%), depression (31.8% vs. 22.8%–25.0%), and PTSD (11.4% vs. 3%). The higher prevalence of anxiety, depression, and PTSD may be explained by the pervading climate of uncertainty generated with the advancing pandemic, limited signs of a workable vaccine, increased workload, lack of social support, and an intense fear of family transmission [86,87,88]. According to a recent Chinese report of 14,825 healthcare workers, depressive symptoms and PTSD were more common among HCWs with lower levels of social support and longer daily working hours (>12 h/day) [88]. We found a lower prevalence of insomnia compared to a previous analysis [14] (27.8% vs. 34.4%) despite having a greater number of studies in the current analysis (11 vs. 5). This may be due to the variance in the cut-off values (>14 vs. >8) of the ISI used by the recent studies, which were included in this updated meta-analysis [32,44,63,64]. Moreover, the direction of the etiological relationships between psychological morbidities and insomnia remains complex; for instance, anxiety disorders precede insomnia in nearly 70% of the cases [89]. Therefore, it is likely that significant changes in sleep architecture among HCWs will occur later and will be observed by prospective studies. In concordance with previous studies, our subgroup analysis by gender revealed that females had a higher prevalence of anxiety and depression compared to males [14,15,27,28,89,90,91,92]. The current meta-analysis found higher levels of anxiety and depression among nurses compared to doctors, which may be because nurses have closer and prolonged contact with patients compared to doctors [3,93]. We investigated the prevalence of anxiety and depression by risk groups and found higher levels of anxiety and depression (as expected) among frontline responders as compared to the second-line workers. All of these results may be partly confounded by the fact that majority of the frontline workers’ group constitutes nurses, who are responsible for providing direct care to the COVID-19 patients and for collecting sputum specimen for virus detection, and tend to be female [3,27,28,94,95,96,97]. This repeats a finding from the SARS outbreak that nurses reported higher anxiety, depression, behavioral problems, and moral injuries (related to death and ethical dilemmas) [3,5,7,14,27,28,92,93,94,95]. These intersecting factors make clear that it is imperative to develop interventions for the most vulnerable population: frontline nurses, who tend to be female and work long hours. Our study reported a higher prevalence of anxiety in other countries compared to China (40.4% vs. 28.5%). The reasons for this may be complex and could be influenced by how other countries are directing their medical resources towards the containment efforts or in treating infected patients rather than providing psychological services.

The higher prevalence of the psychosocial impact of COVID-19 on health care workers found in this study draws more careful attention to educational and policy interventions for this subgroup. Educational and behavioral interventions emphasizing hardiness, social support, positive thinking, a sense of coherence, and others have been advocated in the literature [96,97,98]. The role of self-care is also highlighted by some researchers [98,99,100]. Positive traumatic growth (a positive approach to the management of complex traumas) among health care workers has gained special attention, and interventions to build this have been suggested [96,99,100]. Some strategies, such as mindfulness interventions, can be instituted quickly to promote healthcare workers’ mental health [97,98,99]. Organizational support is vital at the policy level and may need sufficient lead time to be enacted [97,98,99]. Work-based interventions, such as curtailing hours of work, having buddy support systems, having listening sessions between administration and health care functionaries, increasing coverage of tele counseling through employee assistance programs, having mental health consultants available to staff through telehealth, and other such measures, can go a long way in reducing the adverse psychosocial impact caused by COVID-19 [98,99].

### 4.1. Quality of Evidence

Compared to the previous reviews and meta-analyses, the current meta-analysis provides the most extensive evidence with a much bigger sample size of 79,437 participants. Although all studies included in our meta-analysis were cross-sectional, they were of high and medium quality. We performed subgroup analyses to account for the potential sources of heterogeneity and to identify additional vulnerabilities. Additionally, we investigated the potential for publication bias across all studies.

### 4.2. Study Limitations

There are a few limitations that merit discussion. First is the presence of heterogeneity across studies in terms of the survey tool and cut-off scores. Additionally, threshold criteria for defining levels of outcomes varied across studies; for example, some studies reported results as mild, moderate, moderate-severe, and severe, while others reported outcomes as mild, moderate, and severe. This may affect our subgroup analysis by severity. Second, data provided by the studies included in this meta-analysis depend on the self-reported psychological outcomes as recorded through assessment tools. Thus, there may be uncertainty related to the actual psychological illness or diagnosis. Third, sampling bias may exist (although lower than the previous meta-analysis), because nearly 48% (31/65) of the studies were conducted in China. This may also limit the generalizability of the results. Fourth, all studies included in this meta-analysis were cross-sectional, which only provided a snapshot of the existing situation with no exploration of longitudinal aspects. Last, we expect to have a language bias in the study because only studies published in the English language were included.

### 4.3. Research and Clinical Implications

The findings of this meta-analysis highlight the need to develop psychological interventions to promote the post-traumatic growth among HCWs. Higher prevalence estimates of stress provided by this study have important implications for developing early interventions to prevent PTSD, which may be higher in the repairing phases of the pandemic. Mental health and well-being interventions, such as education on coping techniques, online wellness activities, fostering post-traumatic growth, and opening channels for assistance in early signs of PTSS (Posttraumatic Stress Syndrome) before they manifest to PTSD, are essential.

## 5. Conclusions

This article represents, to our knowledge, the most extensive meta-analysis to assess the psychological impact of COVID-19 among HCWs. This meta-analysis provides additional evidence to the higher psychological impact among HCWs, particularly those who are female, nurses, and frontline responders. Furthermore, this adds a valuable insight to the existing meta-analysis findings by highlighting a significant difference in the psychological impact across frontline and second-line workers.

## Figures and Tables

**Table 1:** Subgroup analyses of anxiety across different categories.

**Overall**		**Number of Studies**	**Proportion (%)**	**95% CI**	**I^2^**	***p* Value**	**References**
Anxiety prevalence		46	34.4%	29.5–39.7	99.1%	<0.0001	[6,16,17,18,19,20,21,23,24,25,27,28,29,30,31,32,34,35,41,42,43,44,45,47,48,49,50,52,54,55,57,61,63,64,65,66,67,68,69,70,71,72,73,74,79,80,81]
**Subgroup Analysis**							
**Categories**	**Subgroups**	**Number of Studies**	**Proportion (%)**	**95% CI**	**I^2^**	***p* Value**	**References**
Quality	Good	22	31.2%	24.5–38.7	99.2%	<0.0001	[16,17,18,19,24,25,28,31,32,34,41,42,44,54,57,61,63,64,66,68,79,80]
	Medium	24	38.1%	30.7–46.0	99.0%	<0.0001	[6,20,21,23,27,29,30,35,43,47,48,49,50,52,55,65,67,69,70,71,72,73,74,81]
Continents	Asia	34	32.7%	27.1–38.8	99.2%	<0.0001	[6,16,17,18,19,20,21,23,24,25,27,28,29,30,31,32,34,41,42,43,44,48,55,57,61,63,65,68,69,70,71,73,74,79]
	Other	12	39.3%	29.6–49.9	97.8%	<0.0001	[35,47,49,50,52,54,64,66,67,72,80,81]
Countries	China	22	28.5%	22.5–35.4	99.3%	<0.0001	[16,17,18,19,20,23,24,25,27,28,30,31,32,41,42,57,63,68,70,71,73,79]
	Other	24	40.4%	33.2–48.0	98.4%	<0.0001	[6,21,29,34,35,43,44,47,48,49,50,52,54,55,61,64,65,66,67,69,72,74,80,81]
Assessment	GAD	19	36.8%	29.1–45.2	99.1%	<0.0001	[6,16,17,18,25,31,35,41,49,50,61,63,64,65,66,67,70,72,74]
	SAS	9	24.6%	16.1–35.6	99.7%	<0.0001	[23,24,27,28,32,42,57,68,69]
	Other	18	37.1%	29.1–45.9	99.0%	<0.0001	[19,20,21,29,30,34,43,44,45,47,48,52,54,55,71,73,79,80,81]
Gender	Female	7	46.9%	38.6–55.3	84.6%	<0.0001	[16,47,48,49,73,74,80]
	Male	7	44.2%	36.3–52.5	93.2%	<0.0001	[16,47,48,49,73,74,80]
Healthcare Professions	Nurses	8	39.3%	27.5–52.6	98.9%	<0.0001	[16,23,41,42,55,63,66,73]
	Doctors	8	32.5%	21.9–45.2	98.9%	<0.0001	[16,23,41,42,55,63,66,73]
Healthcare Workers	Frontline	5	39.8%	24.1–58.0	98.6%	<0.0001	[16,23,43,44,79]
	Second-line	5	27.1%	15.1–43.7	99.0%	<0.0001	[16,23,43,44,79]
Level of Anxiety	Mild	18	60.3%	53.8–66.4	94.8%	<0.0001	[6,16,23,24,27,28,30,34,35,43,48,49,54,55,57,61,65,68]
	Moderate	18	26.0%	21.4–31.3	95.4%	<0.0001	[6,16,23,24,27,28,30,34,35,43,48,49,54,55,57,61,65,68]
	Severe	18	14.3	11.2–18.1	97.1%	<0.0001	[6,16,23,24,27,28,30,34,35,43,48,49,54,55,57,61,65,68]

CI = Confidence Interval; GAD = Generalized Anxiety Disorder; SAS = Self-rating Anxiety Scale; Good quality score = 7–9; Medium Quality score = 4–6; I^2^ statistic indicates the heterogeneity.

**Table 2:** Subgroup analyses of depression across different categories.

**Overall**		**Number of Studies**	**Proportion (%)**	**95% CI**	**I^2^**	***p* Value**	**References**
Depression prevalence		46	31.8%	26.8–37.2	99.2%	<0.001	[16,17,18,19,20,21,23,25,27,29,30,31,32,33,34,36,39,41,42,44,45,46,47,48,49,50,52,54,55,57,59,61,63,64,65,66,67,68,70,71,72,73,74,78,79,80]
**Subgroup Analysis**							
**Categories**	**Subgroups**	**Number of Studies**	**Proportion (%)**	**95% CI**	**I^2^**	***p* Value**	**References**
Quality	Good	24	35.1%	27.6–43.5	99.5%	<0.001	[16,17,18,19,25,27,31,32,34,39,41,42,44,46,54,57,59,61,63,64,66,68,79,80]
	Medium	22	28.6%	21.6–36.7	97.9%	<0.001	[20,21,23,29,30,33,36,45,47,48,49,50,52,55,65,67,70,71,72,73,74,78]
Continents	Asia	34	30.8%	25.1–37.1	99.4%	<0.001	[16,17,18,19,20,21,23,25,27,29,30,31,32,33,34,39,41,42,44,46,48,55,57,59,61,63,65,68,70,71,73,74,78,79]
	Other	12	35.0%	24.9–46.7	98.1%	<0.001	[36,45,47,49,50,52,54,64,66,67,72,80]
Countries	China	23	33.2%	26.0–41.3	99.4%	<0.001	[16,17,18,19,20,23,25,27,30,31,32,33,39,41,42,46,57,63,70,71,73,79]
	Other	23	30.4%	23.6–38.3	98.7%	<0.001	[21,29,34,36,44,45,47,48,49,50,52,54,55,59,61,64,65,66,67,72,74,78,80]
Assessment	PHQ	25	29.7%	23.1–37.2	99.4%	<0.001	[16,17,18,29,31,33,36,39,41,45,46,49,50,59,61,63,64,65,66,68,70,72,74,78,79]
	Other	21	34.7%	26.8–43.5	98.9%	<0.001	[19,20,21,23,25,27,30,32,34,42,44,47,48,52,54,55,57,67,71,73,80]
Gender	Female	7	43.4%	33.6–53.9	95.8%	<0.001	[16,47,48,59,73,74,80]
	Male	7	40.9%	31.4–51.5	95.5%	<0.001	[16,47,48,59,73,74,80]
Healthcare Professions	Nurses	9	42.4%	30.4–55.4	99.0%	<0.001	[16,23,33,41,42,55,63,66,73]
	Doctors	9	39.1%	27.3–52.2	98.4%	<0.001	[16,23,33,41,42,55,63,66,73]
Healthcare Workers	Frontline	6	23.6%	14.1–36.7	99.1%	<0.001	[16,17,21,23,44,79]
	Second-line	6	19.6%	11.5–31.5	98.8%	<0.001	[16,17,21,23,44,79]
Level of Depression	Mild	17	57.6%	50.0–64.8	97.8%	<0.001	[16,23,27,30,34,36,39,46,48,54,57,59,61,68,70,71,78]
	Moderate	17	27.9%	22.1–34.6	97.9%	<0.001	[16,23,27,30,34,36,39,46,48,54,57,59,61,68,70,71,78]
	Severe	17	10.4%	7.0–14.0	97.8%	<0.001	[16,23,27,30,34,36,39,46,48,54,57,59,61,68,70,71,78]

CI = Confidence Interval; PHQ = Patients Health Questionnaire; Good quality score = 7–9; Medium Quality score = 4–6; I^2^ statistic indicates the heterogeneity.

**Table 3:** Subgroup analyses of stress across different categories.

**Overall**		**Number of Studies**	**Proportion (%)**	**95% CI**	**I^2^**	***p* Value**	**References**
Stress prevalence		17	40.3%	31.4–50.0	99.1%	<0.001	[18,20,21,31,32,34,41,44,48,54,58,60,62,64,66,73,76]
**Subgroup Analysis**							
**Categories**	**Subgroups**	**Number of Studies**	**Proportion (%)**	**95% CI**	**I^2^**	***p* Value**	**References**
Quality	Good	9	37.3%	25.6–50.7	99.4%	<0.001	[18,31,34,41,44,54,60,64,66]
	Medium	8	45.7%	31.2–61.1	98.4%	<0.001	[20,21,32,48,58,62,73,76]
Continents	Asia	14	41.3%	30.9–52.6	99.2%	<0.001	[18,20,21,31,32,34,41,44,48,58,60,62,73,76]
	Other	3	38.8%	20.6–60.8	99.1%	<0.001	[54,64,66]
Countries	China	7	44.2%	30.9–58.4	99.5%	<0.001	[18,20,31,32,41,73,76]
	Other	10	37.1%	25.4–50.5	98.6%	<0.001	[21,34,44,48,54,58,60,62,64,66]
Survey Instrument	PSS	8	61.4%	45.1–75.6	98.6%	<0.001	[20,32,58,60,62,64,73,76]
	DASS	5	17.5%	9.4–30.3	98.5%	<0.001	[21,34,44,48,54]
	Other	4	47.8%	29.3–66.8	99.7%	<0.001	[18,31,41,66]
Level of Stress	Mild	6	25.8%	16.8–37.6	91.2%	<0.001	[34,48,54,58,60,62]
	Moderate	6	52.3%	38.7–65.5	95.8%	<0.001	[34,48,54,58,60,62]
	Severe	6	18.9%	11.9–28.9	93.4%	<0.001	[34,48,54,58,60,62]

CI = Confidence Interval; PSS = Perceived Stress Scale; DASS = Depression Anxiety Stress Scale; Good quality score = 7–9; Medium Quality score = 4–6; I^2^ statistic indicates the heterogeneity.

## Appendix A

**Table A1:** Database search strategies for psychological impact of COVID-19 among healthcare workers (search date: 27 July 2020).

Database: Ovid MEDLINE(R) and Epub Ahead of Print, In-Process and Other Non-Indexed Citations and Daily <1946 to 27 July 2020> Search Strategy: ------------------------------------------------------------------------------------------------------------------- (2019nCoV or 2019-nCoV or coronavirus or coronavirinae or (corona adj3 (virinae or virus)) or “Corona virinae19” or “Corona virinae2019” or “corona virus19” or “corona virus2019” or Coronavirinae19 or Coronavirinae2019 or coronavirus19 or coronavirus2019 or covid19 or COVID-19 or SARS-CoV-2 or “Severe Acute Respiratory Syndrome Corona virus 2” or “Severe Acute Respiratory Syndrome Coronavirus 2”).ti,ab,kw. [covid-19 keywords] (46270) coronavirus/or Coronavirus Infections/[covid-19 MeSH] (19104) or/1–2 [covid-19 set] (48733) mental health/or mental fatigue/or Affective Symptoms/or psychological distress/[Mental health MeSH] (53257) (emotional disturbanc* or affective symptom* or Alexithymia* or ((mental or psychological) adj3 (fatigue or health or status or distress or well-being)) or psychosocial).ti,ab,kw. [mental health keywords] (283768) or/4–5 [mental health set] (305532) Stress, Psychological/or occupational stress/or compassion fatigue/or burnout, psychological/or burnout, professional/[stress MeSH] (131108) (stress* or “adaptation syndrome” or (caregiver adj4 (burden or fatigue)) or “compassion fatigue” or “reality shock” or “social defeat”).ti,ab,kw. [stress keywords] (842732) or/7–8 [stress set] (897231) Depression/or anhedonia/[depression MeSH] (119688) (depression or depressed or anhedonia or dysphoria or dysthymia or melancholia or sadness).ti,ab,kw. [depression keywords] (404119) or/10–11 [depression set] (436174) anxiety/or catastrophization/[anxiety MeSH] (81955) (anxiety or Catastrophiz* or hypervigilan* or nervousness).ti,ab,kw. [anxiety keywords] (195877) or/13–14 [anxiety set] (218337) “Sleep Initiation and Maintenance Disorders”/[insomnia MeSH] (13134) (drowsiness or dyssomnia * or hypersomnia * or insomnia * or parasomnia *

The asterisk (“*”) used in the search string serves as a truncation operator.

## Appendix B

**Table A2:** Methodological quality assessment of included studies using the National Institutes of Health (NIH) tool.

Author/Year	Q1	Q2	Q3	Q4	Q5	Q6	Q7	Q8	Q9	Q10	Q11	Q12	Q13	Q14	Final Quality Score	Rating
Aksoy and Kocak et al., 2020 [81]	Y	Y	NR	N	N	Y	N	N	Y	NA	Y	NA	NA	N	5	Medium
Alshekaili et al., 2020 [44]	Y	Y	Y	Y	N	Y	Y	Y	Y	NA	Y	NA	NA	Y	8	Good
Amerio et al., 2020 [45]	Y	Y	N	N	N	Y	Y	Y	Y	NA	N	NA	NA	N	6	Medium
Amin et al., 2020 [37]	Y	Y	Y	N	N	Y	N	N	Y	NA	Y	NA	NA	N	5	Medium
An et al., 2020 [46]	Y	Y	NR	Y	Y	Y	Y	Y	Y	NA	Y	NA	NA	N	9	Good
Cai et al., 2020 [79]	Y	Y	NR	Y	Y	Y	Y	Y	Y	NA	Y	NA	NA	N	9	Good
Caliskan et al., 2020 [47]	Y	Y	NR	N	N	Y	Y	N	Y	NA	Y	NA	NA	N	6	Medium
Cao et al., 2020 [33]	Y	Y	Y	N	N	Y	N	N	Y	NA	Y	NA	NA	N	6	Medium
Chatterjee et al., 2020 [48]	Y	Y	NR	N	N	Y	N	Y	Y	NA	Y	NA	NA	N	6	Medium
Chen et al., 2020 [30]	Y	Y	Y	N	N	Y	N	N	NR	NA	Y	NA	NA	N	5	Medium
Chew et al., 2020 [34]	Y	Y	Y	N	Y	Y	Y	N	Y	NA	Y	NA	NA	N	8	Good
Chung and Yeung et al., 2020 [78]	Y	Y	NR	N	N	Y	N	N	Y	NA	Y	NA	NA	N	5	Medium
Civantos et al., 2020 [49]	Y	Y	N	N	N	Y	Y	N	Y	NA	Y	NA	NA	N	6	Medium
Consolo et al., 2020 [35]	Y	Y	N	N	N	Y	Y	N	Y	NA	Y	NA	NA	N	6	Medium
Corbett et al., 2020 [50]	Y	Y	N	N	N	Y	Y	N	Y	NA	Y	NA	NA	N	6	Medium
Dai et al., 2020 [51]	Y	Y	Y	N	N	Y	Y	N	Y	NA	N	NA	NA	N	6	Medium
Dal’Bosco et al., 2020 [52]	Y	Y	N	N	N	Y	Y	N	Y	NA	Y	NA	NA	N	6	Medium
Dong et al., 2020 [53]	Y	Y	NR	Y	Y	Y	Y	N	Y	NA	Y	NA	NA	N	8	Good
Du et al., 2020 [20]	Y	Y	NR	N	N	Y	Y	N	Y	NA	Y	NA	NA	N	6	Medium
Elbay et al., 2020 [54]	Y	Y	NR	Y	Y	Y	Y	N	Y	NA	Y	NA	NA	N	8	Good
Guiroy et al., 2020 [36]	Y	Y	N	N	N	Y	Y	N	Y	NA	Y	NA	NA	N	6	Medium
Guo et al., 2020 [23]	Y	Y	NR	N	N	Y	Y	N	Y	NA	Y	NA	NA	N	6	Medium
Hassannia et al., 2020 [55]	Y	Y	Y	N	N	Y	Y	N	Y	NA	N	NA	NA	N	6	Medium
Hawari et al., 2020 [56]	Y	Y	NR	N	N	Y	Y	N	Y	NA	Y	NA	NA	N	6	Medium
Hu et al., 2020 [57]	Y	Y	Y	Y	Y	Y	Y	N	Y	NA	Y	NA	NA	N	9	Good
Huang and Zhao et al., 2020 [25]	Y	Y	Y	Y	Y	Y	Y	N	Y	NA	Y	NA	NA	N	9	Good
Huang et al., 2020 [24]	Y	Y	Y	Y	Y	Y	Y	N	Y	NA	Y	NA	NA	N	9	Good
Jahrami et al., 2020 [58]	Y	Y	Y	N	N	Y	N	N	Y	NA	Y	NA	NA	N	6	Medium
Kang et al., 2020 [39]	Y	Y	NR	Y	Y	Y	Y	Y	Y	NA	Y	NA	NA	N	9	Good
Kaveh et al., 2020 [43]	Y	Y	NR	N	N	Y	Y	N	Y	NA	Y	NA	NA	N	6	Medium
Khanna et al., 2020 [59]	Y	Y	NR	Y	Y	Y	Y	N	Y	NA	Y	NA	NA	N	8	Good
Koksal et al., 2020 [80]	Y	Y	NR	Y	Y	Y	Y	N	Y	NA	Y	NA	NA	N	8	Good
Lai et al., 2020 [16]	Y	Y	Y	Y	Y	Y	Y	N	Y	NA	Y	NA	NA	N	9	Good
Li et al., 2020 [31]	Y	Y	Y	Y	N	Y	Y	N	Y	NA	Y	NA	NA	N	8	Good
Liu et al., 2020 [27]	Y	Y	NR	N	N	Y	Y	N	Y	NA	Y	NA	NA	N	6	Medium
Liu et al., 2020 [28]	Y	Y	Y	Y	Y	Y	Y	N	Y	NA	Y	NA	NA	N	9	Good
Lu et al., 2020 [19]	Y	Y	Y	Y	Y	Y	Y	N	Y	NA	Y	NA	NA	N	9	Good
Nair et al., 2020 [60]	Y	Y	NR	Y	N	Y	Y	N	Y	NA	Y	NA	NA	N	7	Good
Naser et al., 2020 [61]	Y	Y	NR	Y	N	Y	Y	Y	Y	NA	Y	NA	NA	N	8	Good
Podder et al., 2020 [62]	Y	Y	NR	N	N	Y	Y	N	Y	NA	Y	NA	NA	N	6	Medium
Qi et al., 2020 [26]	Y	Y	Y	Y	Y	Y	N	N	Y	NA	Y	NA	NA	N	8	Good
Que et al., 2020 [63]	Y	Y	NR	Y	Y	Y	Y	Y	Y	NA	Y	NA	NA	N	9	Good
Rossi et al., 2020 [64]	Y	Y	NR	Y	N	Y	Y	Y	Y	NA	Y	NA	NA	Y	9	Good
Salman et al., 2020 [65]	Y	Y	NR	N	N	Y	Y	N	Y	NA	Y	NA	NA	N	6	Medium
Shacham et al., 2020 [38]	Y	Y	NR	N	N	Y	Y	N	Y	NA	Y	NA	NA	N	6	Medium
Shechter et al., 2020 [66]	Y	Y	Y	Y	Y	Y	Y	N	Y	NA	Y	NA	NA	N	9	Good
Stojanov et al., 2020 [67]	Y	Y	NR	N	N	Y	Y	N	Y	NA	Y	NA	NA	N	6	Medium
Sun et al., 2020 [77]	Y	Y	NR	N	N	Y	Y	N	Y	NA	Y	NA	NA	N	6	Medium
Tan et al., 2020 [21]	Y	Y	Y	N	N	Y	Y	N	Y	NA	Y	NA	NA	N	6	Medium
Temsah et al., 2020 [6]	Y	Y	Y	N	N	Y	Y	N	N	NA	Y	NA	NA	N	6	Medium
Teng et al., 2020 [68]	Y	Y	N	Y	Y	Y	N	Y	Y	NA	Y	NA	NA	Y	9	Good
Thapa et al., 2020 [69]	Y	Y	NR	N	N	Y	N	N	Y	NA	Y	NA	NA	N	5	Medium
Tu et al., 2020 [70]	Y	Y	Y	N	N	Y	Y	N	N	NA	Y	NA	NA	N	6	Medium
Wang et al., 2020 [32]	Y	Y	Y	Y	N	Y	Y	N	Y	NA	Y	NA	NA	N	8	Good
Wang et al., 2020 [71]	Y	Y	Y	N	N	Y	Y	N	N	NA	Y	NA	NA	N	6	Medium
Weilenmann et al., 2020 [72]	Y	Y	NR	N	N	Y	Y	N	Y	NA	Y	NA	NA	N	6	Medium
Xiao et al., 2020 [73]	Y	Y	NR	N	N	Y	N	Y	Y	NA	Y	NA	NA	N	6	Medium
Yang et al., 2020 [74]	Y	Y	Y	N	N	Y	N	N	Y	NA	Y	NA	NA	N	6	Medium
Yin et al., 2020 [75]	Y	Y	Y	Y	Y	Y	Y	N	Y	NA	Y	NA	NA	N	9	Good
Zhan et al., 2020 [76]	Y	Y	Y	N	N	Y	Y	N	N	NA	Y	NA	NA	N	6	Medium
Zhang et al., 2020 [17]	Y	Y	NR	Y	Y	Y	Y	Y	Y	NA	Y	NA	NA	N	9	Good
Zhang et al., 2020 [18]	Y	Y	Y	Y	Y	Y	Y	N	Y	NA	Y	NA	NA	N	9	Good
Zhang et al., 2020 [29]	Y	Y	NR	N	N	Y	Y	N	Y	NA	Y	NA	NA	N	6	Medium
Zhu et al., 2020 [41]	Y	Y	Y	Y	Y	Y	Y	N	Y	NA	Y	NA	NA	N	9	Good
Zhu et al., 2020 [42]	Y	Y	Y	Y	N	Y	Y	N	Y	NA	Y	NA	NA	N	8	Good

Y: Yes, N: No, NR: Not reported, NA: Not applicable. (Q1. Was the research question or objective in this paper clearly stated? Q2. Was the study population clearly specified and defined? Q3. Was the participation rate of eligible persons at least 50%? Q4. Were all the subjects selected or recruited from the same or similar populations (including the same time period)? Were inclusion and exclusion criteria for being in the study prespecified and applied uniformly to all participants? Q5. Was a sample size justification, power description, or variance and effect estimates provided? Q6. For the analyses in this paper, were the exposure(s) of interest measured prior to the outcome(s) being measured? Q7. Was the timeframe sufficient so that one could reasonably expect to see an association between exposure and outcome if it existed? Q8. For exposures that can vary in amount or level, did the study examine different levels of the exposure as related to the outcome (e.g., categories of exposure, or exposure measured as continuous variable)? Q9. Were the exposure measures (independent variables) clearly defined, valid, reliable, and implemented consistently across all study participants? Q10. Was the exposure(s) assessed more than once over time? Q11. Were the outcome measures (dependent variables) clearly defined, valid, reliable, and implemented consistently across all study participants? Q12. Were the outcome assessors blinded to the exposure status of participants? Q13. Was loss to follow-up (response rate) after baseline 20% or less? Q14. Were key potential confounding variables measured and adjusted statistically for their impact on the relationship between exposure(s) and outcome(s)? Rating—Good, Medium or Poor), Good = (7–9 yes); Medium = (4–6 yes).)

## Appendix C

**Table A3:** Characteristics of studies meeting the search inclusion criteria.

Author/Year	Sample Size	Country	Health Care Workers		Male (%)	Survey Tool	Cut-Off	Outcomes (%) (n)					
			Physician (%)	Nurses (%)				Depression	Anxiety	Insomnia	Stress	PTSD ^a^	Distress
Aksoy and Kocak et al., 2020 [81]	758	Turkey	0.0	100.0	7.0	STAI ^y^	NA ^ae^	NA	36.3 (275)	NA	NA	NA	NA
Alshekaili et al., 2020 [44]	1139	Oman	33.7	39.4	20.0	DASS ^t^ -21(D ^aa^) DASS ^t^ -21(A ^ab^) DASS ^t^ -21(S ^ac^) ISI ^d^	≥10 ≥8 ≥16 ≥14	32.3 (368)	34.1 (388)	18.5 (211)	23.8 (271)	NA	NA
Amerio et al., 2020 [45]	131	Italy	NA	NA	51.9	PHQ ^c^ -9	≥10	39.3 (30)	NA	NA	NA	NA	NA
Amin et al., 2020 [37]	250	Pakistan	49.2	30.4	63.6	PGWBI ^l^	≥30	NA	NA	NA	NA	NA	72.4 (181)
An et al., 2020 [46]	1103	China	0.0	100.0	9.2	PHQ ^c^ -9	≥5	43.6 (481)	NA	NA	NA	NA	NA
Cai et al., 2020 [79]	2346	China	NA	NA	29.9	PHQ ^c^ -9 BAI ^m^ ISI ^d^	≥10 ≥15 ≥9	12.23 (287)	11.6 (271)	38.4 (902)	NA	NA	NA
Caliskan et al., 2020 [47]	290	Turkey	100.0	NA	61.7	HADS ^k^ -D HADS ^k^ -A	≥7 ≥10	62.1 (180)	35.5 (103)	NA	NA	NA	NA
Cao et al., 2020 [33]	37	China	16.0	19.0	21.6	PHQ ^c^ -9	≥10	18.9 (7)	NA	NA	NA	NA	NA
Chatterjee et al., 2020 [48]	152	India	NA	NA	78.3	DASS ^t^ -21	NA	34.9 (53)	39.5 (60)	NA	32.9 (50)	NA	NA
Chen et al., 2020 [30]	105	China	NA	NA	9.5	SAS ^r^ SDS ^z^	≥50 ≥50	29.5 (31)	18.1 (19)	NA	NA	NA	NA
Chew et al., 2020 [34]	906	Singapore and India	NA	NA	NA	DASS ^t^ -21(D ^aa^) DASS ^t^ -21(A ^ab^) DASS ^t^ -21(S ^ac^) IES ^i^ -R	>9 >7 >14 ≥24	21.4 (96)	31.5 (142)	NA	10.3 (47)	14.8 (67)	NA
Chung and Yeung et al., 2020 [78]	69	Hong Kong	4.4	34.8	NA	PHQ ^c^ -9	≥10	49.3 (34)	NA	NA	NA	NA	NA
Civantos et al., 2020 [49]	349	USA	52.7	NA	60.7	GAD ^b^ -7 PHQ ^c^ -2 IES ^i^	≥10 ≥3 ≥27	10.6 (37)	47.9 (167)	NA	NA	NA	60.2 (210)
Consolo et al., 2020 [35]	356	Italy	NA	NA	60.4	GAD ^b^ -7	≥5	NA	57.0 (204)	NA	NA	NA	NA
Corbett et al., 2020 [50]	240	Dublin Ireland	15.0	36.25	9.2	GAD ^b^ -7 PHQ ^c^ -9	≥10 ≥10	20.3 (49)	21.0 (51)	NA	NA	NA	NA
Dai et al., 2020 [51]	4357	China	32.6	53.8	23.5	GHQ ^e^ -12	≥3	NA	NA	NA	NA	NA	39.1 (1704)
Dal’Bosco et al., 2020 [52]	88	South America	NA	NA	10.2	HAD ^k^	≥3	25.0 (22)	48.9 (43)	NA	NA	NA	NA
Dong et al., 2020 [53]	4618	China	24.6	62.7	16.3	HEI ^q^	≥8	NA	NA	NA	NA	NA	24.2 (1118)
Du et al., 2020 [20]	134	China	35.1	41.0	39.6	BDI ^o^ -II BAI ^m^ PSS ^j^	≥14 ≥8 ≥14	12.7 (17)	20.1 (28)	NA	59.0 (79)	NA	NA
Elbay et al., 2020 [54]	442	Turkey	NA	NA	43.2	DASS ^t^ -21(D ^aa^) DASS ^t^ -21(A ^ab^) DASS ^t^ -21(S ^ac^)	>9 >7 >14	64.7 (286)	51.6 (224)	NA	41.2 (182)	NA	NA
Guiroy et al., 2020 [36]	204	Latin America ^ad^	100.0	NA	96.6	PHQ ^c^ -9	≥10	100 (204)	NA	NA	NA	NA	NA
Guo et al., 2020 [23]	11,118	China	30.28	53.07	25.2	SAS ^r^ SDS ^z^	≥50 ≥50	31.5 (3497)	17.5 (1940)	NA	NA	NA	40.7 (4530)
Hassannia et al., 2020 [55]	487	Iran	26.08	21.56	NA	HADS ^k^ -D ^aa^ HADS ^k^ -A ^ab^	≥8 ≥8	48.3 (235)	62.8 (306)	NA	NA	NA	NA
Hawari et al., 2020 [56]	1006	Jordan	13.02	63.02	44.7	K6 ^ag^	≥11	NA	NA	NA	NA	NA	96.5 (971)
Hu et al., 2020 [57]	2101	China	NA	100.00	12.4	SAS ^r^ ZSDS ^v^	≥50 ≥60	42.0 (878)	40.0 (833)	NA	NA	NA	41.5 (835)
Huang and Zhao et al., 2020 [25]	2250	China	NA	NA	NA	GAD ^b^ -7 CES-D ^s^ PSQI ^p^	≥9 ≥28 ≥7	19.8 (446)	35.6 (802)	23.6 (531)	NA	NA	NA
Huang et al., 2020 [24]	230	China	30.4	69.6	18.7	SAS ^r^ PTSD-SS	≥50 ≥50	NA	23.0 (53)	NA	NA	27.4 (63)	NA
Jahrami et al., 2020 [58]	257	Bahrain	31.1	46.3	30.0	PSQI ^p^ PSS ^j^	≥5 ≥14	NA	NA	NA	100.0 (257)	NA	NA
Kang et al., 2020 [39]	994	China	18.4	81.6	14.5	PHQ ^c^ -9 GAD ^b^ -7 ISI ^d^ IES ^i^ -R	≥5 ≥5 ≥8 ≥9	63.0 (627)	NA	NA	NA	NA	NA
Kaveh et al., 2020 [43]	1038	Iran	20.6	63.3	12.4	BAI ^m^	≥7	NA	100.0 (1038)	NA	NA	NA	NA
Khanna et al., 2020 [59]	2355	India	NA	NA	56.6	PHQ ^c^ -9	≥4	32.6 (765)	NA	NA	NA	NA	NA
Koksal et al., 2020 [80]	702	Turkey	NA	48.3	30.0	HADS ^k^ -D ^aa^ HADS ^k^ -A ^ab^	≥7 ≥10	36.9 (259)	57.5 (404)	NA	NA	NA	NA
Lai et al., 2020 [16]	1257	China	39.2	60.8	23.3	PHQ ^c^ -9 GAD ^b^ -7 ISI ^d^ IES ^i^ -R	≥5 ≥5 ≥8 ≥9	50.4 (634)	44.6 (560)	34 (427)	NA	NA	71.5 (899)
Li et al., 2020 [31]	4369	China	13.3	77.4	0.0	IES ^i^ –R PHQ ^c^ -9 GAD ^b^ -7	≥33 ≥10 ≥8	14.2 (621)	25.2 (1101)	NA	31.6 (1382)	NA	NA
Liu et al., 2020 [27]	512	China	NA	NA	15.4	SAS ^r^	≥50	NA	12.5 (64)	NA	NA	NA	NA
Liu et al., 2020 [28]	4679	China	39.6	60.4	17.7	SAS ^r^ SDS ^z^ SRQ ^x^ -20	≥50 ≥50 ≥7	34.6 (1619)	16.0 (749)	NA	NA	NA	15.9 (744)
Lu et al., 2020 [19]	2299	China	88.8	NA	22.4	HADS ^k^ -D ^aa^ HADS ^k^ -A ^ab^	≥7 ≥7	11.7 (268)	24.7 (569)	NA	NA	NA	NA
Nair et al., 2020 [60]	586	India	NA	NA	53.1	CPDI ^w^ PSS ^j^	≥28 ≥13	NA	NA	NA	100.0 (586)	NA	52.0 (304)
Naser et al., 2020 [61]	1163	Jordan	48.2	13.0	43.9	GAD ^b^ -7 PHQ ^c^ -9	≥4 ≥5	78.0 (907)	71.0 (823)	NA	NA	NA	NA
Podder et al., 2020 [62]	384	India	NA	NA	55.5	PSS ^j^ -10	≥13	NA	NA	NA	100 (384)	NA	NA
Qi et al., 2020 [26]	1306	China	NA	NA	19.6	PSQI ^p^ AIS ^f^	>7 >6	NA	NA	45.5 (594)	NA	NA	NA
Que et al., 2020 [63]	2285	China	37.6	9.1	30.9	GAD ^b^ -7 PHQ ^c^ ISI ^d^	≥10 ≥10 ≥15	44.4 (1014)	46.0 (1052)	28.8 (657)	NA	NA	NA
Rossi et al., 2020 [64]	1379	Italy	37.64	34.23	22.8	GPS PHQ ^c^ -9 GAD ^b^ -7 ISI ^d^ -7 PSS ^j^ -10	≥3 ≥15 ≥15 ≥22 NA	24.73 (341)	19.8 (273)	8.3 (114)	21.9 (302)	49.4 (681)	NA
Salman et al., 2020 [65]	398	Pakistan	51.5	33.4	46.0	GAD ^b^ -7 PHQ ^c^ -9	≥10 ≥10	21.9 (87)	21.4 (85)	NA	NA	NA	NA
Shacham et al., 2020 [38]	338	Israel	NA	NA	41.4	K6 ^ag^	≥19	NA	NA	NA	NA	NA	11.5 (39)
Shechter et al., 2020 [66]	657	USA	28.8	47.6	21.8	PC-PTSD ^u^ PHQ ^c^ -2 GAD ^b^ -2	≥3 ≥3 ≥3	48.0 (313)	33.0 (215)	NA	57.0 (371)	NA	NA
Stojanov et al., 2020 [67]	201	Serbia	NA	100.0	34.3	GAD ^b^ -7 ZSDS ^v^	≥5 ≥50	30.8(62)	48.2 (97)	NA	NA	NA	NA
Sun et al.,2020 [77]	320	China	NA	NA	NA	PCL ^af^ -5	≥33	NA	NA	NA	NA	4.4 (14)	NA
Tan et al., 2020 [21]	470	Singapore	28.7	34.3	31.7	DASS ^t^ -21(D ^aa^) DASS ^t^ -21(A ^ab^) DASS ^t^ -21(S ^ac^) IES ^i^ -R	>9 >7 ≥14 ≥24	8.9 (42)	14.5 (68)	NA	6.6 (31)	7.7 (36)	NA
Temsah et al., 2020 [6]	582	Saudi Arabian	18.6	62.4	24.9	GAD ^b^ -7	≥5	NA	100.0 (582)	NA	NA	NA	NA
Teng et al., 2020 [68]	398	China	NA	NA	24.1	PHQ ^c^ -9 SAS ^r^	≥5 ≥50	35.9 (143)	14.1 (56)	NA	NA	NA	NA
Thapa et al., 2020 [69]	100	Nepal	9.0	62.0	22.0	SAS ^r^	≥45	NA	34.0 (34)	NA	NA	NA	NA
Tu et al., 2020 [70]	100	China	NA	100.0	0.0	GAD ^b^ -7 PHQ ^c^ -9 PSQI ^p^	≥4 ≥4 ≥7	46.0 (46)	40.0 (40)	NA	NA	NA	NA
Wang et al., 2020 [71]	1045	China	14.3	74.0	14.2	HADS ^k^ -D ^aa^ HADS ^k^ -A ^ab^ PSS ^j^ -14 ISI ^d^	≥8 ≥8 ≥14 ≥14	53.0 (554)	67.8 (708)	10.4 (109)	21.0 (223)	NA	NA
Weilenmann et al., 2020 [72]	1410	Switzerland	60.8	39.2	33.8	GAD ^b^ -7 PHQ ^c^ -9	≥10 ≥10	20.7 (292)	25.9 (365)	NA	NA	NA	NA
Xiao et al., 2020 [73]	958	China	39.5	37.5	32.8	HADS ^k^ -D ^aa^ HADS ^k^ -A ^ab^	≥8 ≥8	57.3 (549)	54.2 (518)	NA	NA	NA	NA
Yang et al., 2020 [74]	65	South Korea	NA	NA	52.3	GAD ^b^ -7 PHQ ^c^ -9	≥5 ≥10	18.5 (12)	32.3 (21)	NA	NA	NA	NA
Yin et al., 2020 [75]	371	China	18.1	71.2	38.5	PCL ^af^ -5	≥33	NA	NA	NA	NA	3.8 (14)	NA
Zhan et al., 2020 [76]	1794	China	NA	100.0	3.0	AIS ^f^ FS ^g^ 14 CPSS ^h^	≥6 ≥7 ≥25	NA	NA	52.8 (948)	44.0 (789)	NA	NA
Zhang et al., 2020 [17]	2182	China	31.2	11.3	35.8	ISI ^d^ SCL ^n^ -90-R PHQ ^c^ -2 GAD ^b^ -2	>8 ≥2 ≥3 ≥3	10.6 (232)	10.4 (228)	33.9 (739)	NA	NA	NA
Zhang et al., 2020 [18]	1563	China	29.0	62.9	17.3	ISI ^d^ PHQ ^c^ -9 GAD ^b^ -7 IES ^i^ -R	≥8 ≥5 ≥5 ≥9	50.7 (792)	44.7 (699)	36.1 (564)	73.4 (1147)	NA	NA
Zhang et al., 2020 [29]	304	Iran	NA	NA	NA	PHQ ^c^ -4 K6 ^ag^	NA NA	20.6 (63)	28.0 (85)	NA	NA	NA	20.1 (61)
Zhu et al., 2020 [41]	5062	China	19.8	67.5	15.0	IES ^i^ -R PHQ ^c^ 9 GAD ^b^ -7	≥33 ≥10 ≥8	13.5 (681)	24.1 (1218)	NA	29.8 (1509)	NA	NA
Zhu et al., 2020 [42]	165	China	47.9	52.1	17.0	SAS ^r^ SDS ^z^	≥50 ≥50	44.2 (73)	20.0 (33)	NA	NA	NA	NA

^a^ PTSD: Post-traumatic stress syndrome, ^b^ GAD: Generalized anxiety disorder scale, ^c^ PHQ: Patient health questionnaire, ^d^ ISI: Insomnia severity index, ^e^ GHQ: General health questionnaire, ^f^ AIS: Athens insomnia scale, ^g^ FS: Fatigue scale, ^h^ CPSS: Chinese perceived stress scale, ^i^ IES: Impact of event scale, ^j^ PSS: Perceived stress scale, ^k^ HAD: Hospital anxiety depression scale, ^l^ PGWBI: Psychological General Well-Being Index, ^m^ BAI: Beck anxiety Inventory, ^n^ SCL-19-R: Symptom checklist, ^o^ BDI: Beck depression inventory, ^p^ PSQI: Pittsburgh sleep quality index, ^q^ HEI: Huaxi emotional distress index, ^r^ SAS: Self-rating anxiety scale, ^s^ CES-D: The center for epidemiology scale for depression, ^t^ DASS: Depression, anxiety, stress scales, ^u^ PC-PTSD: Primary care PTSD Screen, ^v^ Zsds: Zung self-rating depression scale, ^w^ CPDI: COVID-19 Peri traumatic distress index, ^x^ SRQ: Self reporting questionnaire, ^y^ STAI: State-trait anxiety inventory, ^z^ SDS: Self-rating depression scale, ^aa^ D: Depression, ^ab^ A: Anxiety, ^ac^ S: Stress, ^ad^ Latin America: Countries included Brazil, Argentina, Chile, and Mexico, ^ae^ NA: Not applicable, ^af^ PCL: Post-traumatic stress disorder checklist, ^ag^ K6—Kessler Phycological Distress Scale.
